# A surgeon led smoking cessation intervention in a head and neck cancer centre

**DOI:** 10.1186/s12913-014-0636-8

**Published:** 2014-12-20

**Authors:** Ming Wei Tang, Richard Oakley, Catherine Dale, Arnie Purushotham, Henrik Møller, Jennifer Elizabeth Gallagher

**Affiliations:** Division of Population and Patient Health, King’s College London Dental Institute at Guy’s, King’s College and St Thomas’ Hospitals, Denmark Hill Campus, London, SE5 9RS UK; Guy’s and St Thomas’ NHS Foundation Trust, London, UK; Guy’s & St Thomas NHS Foundation Trust, Division of Cancer Studies, King’s College London, London, UK; King’s College London, Cancer Epidemiology and Population Health, London, UK

**Keywords:** Smoking cessation, Stop smoking, Head and neck cancer, Oral cancer, Prevention

## Abstract

**Background:**

The government has recognised the role of healthcare professionals in smoking cessation interventions with integrated care pathways for identification and referral of at-risk patients who smoke. Referral for suspected cancers has been suggested as a ‘teachable moment’, whereby individuals are motivated and more likely to adopt risk-reducing behaviours. A head and neck cancer referral clinic could therefore provide opportunities for smoking cessation intervention.

This study aims to pilot a brief smoking cessation intervention during a consultation visit for patients referred with suspected head and neck cancer and evaluate its acceptability and impact.

**Methods:**

A brief script for smoking cessation intervention which included a smoking cessation referral was designed to be delivered to patients attending a rapid access clinic. Patient outcome data was collected by the stop smoking team for patients who accepted the referral. A subset of these patients was also interviewed by telephone; these findings were combined with data provided by the stop smoking services to assess the acceptability and impact of pilot smoking cessation intervention on patients.

**Results:**

In total, 473 new patients attended the clinic during the study period, of whom 102 (22%) were smokers. Of these, 80 (78%) accepted a referral to stop smoking services. A total of 75 (74%) patients were approached subsequently in a telephone survey. Of the 80 newly referred patients, 29 (36%) quit smoking at least temporarily. Another eight patients reduced their smoking or set a quit date (10%), so the experience of attending the clinic and the intervention impacted favourably on almost half of the patients (46%). The patient survey found the intervention to be acceptable for 94% (n = 50) of patients. Qualitative analysis of patient responses revealed five elements which support the acceptability of the intervention.

**Conclusions:**

The findings of this pilot study suggest that discussion of smoking cessation with patients referred for suspected head and neck cancer may have an impact and facilitate the process towards quitting. A possible diagnosis of cancer appears to present a ‘teachable moment’ to encourage positive health behaviour change.

## Background

Cancers in the head and neck region account for about 3% of all cancers diagnosed in England [1]. The incidence of head and neck cancer nationally and in south east London is increasing [2].

The majority of head and neck cancers are squamous cell carcinomas and the main risk factors are tobacco smoking, alcohol consumption, and human papilloma virus infection. In a large international study, tobacco and alcohol together explained 73% of upper aero-digestive tract (UADT) cancer burden, of which almost 29% was due to tobacco alone, less than 1% alcohol alone and 44% by the joint effect of tobacco and alcohol [3]. Smoking was an independent risk factor for head and neck cancer while alcohol consumption in the absence of smoking conferred little or no risk. The joint effect of tobacco and alcohol increased with the number of pack-years and drink-years [4]. Tobacco smoking is the main modifiable risk factor for head and neck cancer and reducing smoking prevalence is one of three key commitments at the heart of the NHS cancer plan [5].

The government has commissioned several white papers [6-9], which provide guidance for tobacco control policy in England. These documents highlight the important role of healthcare professionals in smoking cessation. It is within their capacity to proactively discuss the issue of smoking with patients as it is linked to improved general health. Integrated care pathways must be developed and utilised for identification and referral of at-risk patients who smoke. The NHS stop smoking service plays a key role within this care pathway. Studies have shown that smokers accessing this service are four times more likely to achieve a four-week quit than those without this assistance [10]. Godfrey et al., found NHS smoking cessation services to be a cost-effective life-saving intervention when compared to many other health-care interventions [11]. Although not ideal, there is evidence that cutting down smoking can be an important part of the process of stopping [12,13].

The importance of making every contact count for health has been emphasised in recent health policy [14]. Patients referred via the two week wait route are perceived by the GP as being at high risk of cancer and early intervention will potentially improve their outcome. With the increasing incidence of head and neck cancer, the initial thrust to pilot a disease prevention program stemmed from the South East London Tumour Working Group. The impact of a smoking cessation intervention is plausibly translatable to patients at risk of other smoking related illnesses.

Events such as attending a head and neck cancer diagnosis service have been suggested to be a ‘teachable moment’ whereby individuals may become motivated to adopt risk-reducing behaviours. Several studies have shown high rates of smoking cessation amongst patients with newly diagnosed head and neck cancer [15,16]. This finding may be explained using the health belief model whereby patients perceive a susceptibility to head and neck cancer and its threat, which may be a ‘cue to action’ because the benefit of quitting smoking is evident [17].

A teachable moment in the health care setting may be exploited to encourage smoking cessation in patients with suspected head and neck cancer [18]. The initial diagnostic clinic provides such an opportunity for healthcare professionals to help modify patient behaviour by giving smoking cessation advice and directing patients towards existing smoking cessation services. Valid concerns about such an intervention include whether the intervention is effective in contributing to smoking cessation, and whether the intervention may be unduly stressful at a moment of anxiety and concern [19].

The aims of this study were to assess the acceptability, and impact, of a brief smoking cessation intervention during the first consultation visit for patients referred with suspected head and neck cancer with a view to informing health policy and action.

## Methods

### The intervention

The study population consisted of all patients who were referred to and attended the head and neck cancer clinic (Mr R Oakley; RO) in 2012 at Guy’s and St Thomas’ NHS Foundation Trust. During the patient’s first consultation visit, RO followed a scripted intervention which was evidence based [20], developed jointly with the stop-smoking co-ordinator (See ‘Intervention protocol’). Patients who were smokers were offered the opportunity to be referred to their local stop smoking team.

#### Intervention protocol

Q: Is there anything you do that may have contributed to the symptoms you have come to see me about today? *If they do not mention smoking*:

Q: Do you think smoking may have contributed to your symptoms? *Following the response, go on to explain that there is a link.*

Q: Have you thought about giving up? I know that it’s not easy to quit smoking so I’d like to refer you to someone who can tell you about what services there are available to help people quit smoking. You can then decide if this help seems likely to work for you. Are you happy to receive this phone call? *If a patient accepts the referral they will be referred via EPR, and given a copy of a letter confirming the referral.*

If the patient declined the referral this was noted and the patient was given an information leaflet about NHS smoking cessation services to take with them.

All referrals were collated by the hospitals stop-smoking coordinator and passed to the appropriate local community team for the patient’s home address.

### Data collection

#### Stop smoking team

The stop smoking team collected data on the outcome of all patients contacted by the team; whether they have made local arrangements to achieve smoking cessation, already quit, accepted or rejected the service.

#### Personal interviews

A survey instrument was designed by the authors to assess patients’ thoughts and opinion on the suitability of the brief smoking cessation intervention during the consultation visit. The questionnaire also included questions on patients’ smoking status. Patients were considered eligible for the post-intervention survey study if they had identified themselves as smokers, accepted a smoking cessation referral and were able to communicate effectively in English.

Eligible patients were sent a letter to inform them of the research group’s intent to undertake a telephone survey and the method of opting out. Patients who did not opt out were contacted by telephone (MW Tang) at least 4 months after the initial consultation appointment. A verbal consent was obtained prior to the interview and patient responses were recorded on a paper survey form by the interviewer. All interviews were conducted by one interviewer to ensure consistency of approach.

### Data analysis

Quantitative results from the telephone survey were linked with the smoking cessation outcome data collected by the stop smoking team. Both sets of data were cross checked for consistency in patient reported outcomes. Outcomes of intervention were divided into four categories:

1. Quit (patients who successfully quit after the intervention regardless of time period);
2. Action (patients who took action in view of quitting eg. set quit date);
3. Motivation (patients who want to quit);
4. Null (patients who could not be contacted or none of the above).

Data were tabulated in a Microsoft Excel spreadsheet and analysed accordingly. The qualitative data were analysed by looking at themes and patterns using inductive reasoning [21]. Descriptive analysis was conducted on the combined data sets to allow a better understanding of the impact of intervention on patient outcome and its acceptability.

Approval for this service evaluation was obtained from the Clinical Governance team at Guy’s and St Thomas NHS Foundation Trust.

## Results

### The intervention

**Figure 1:**
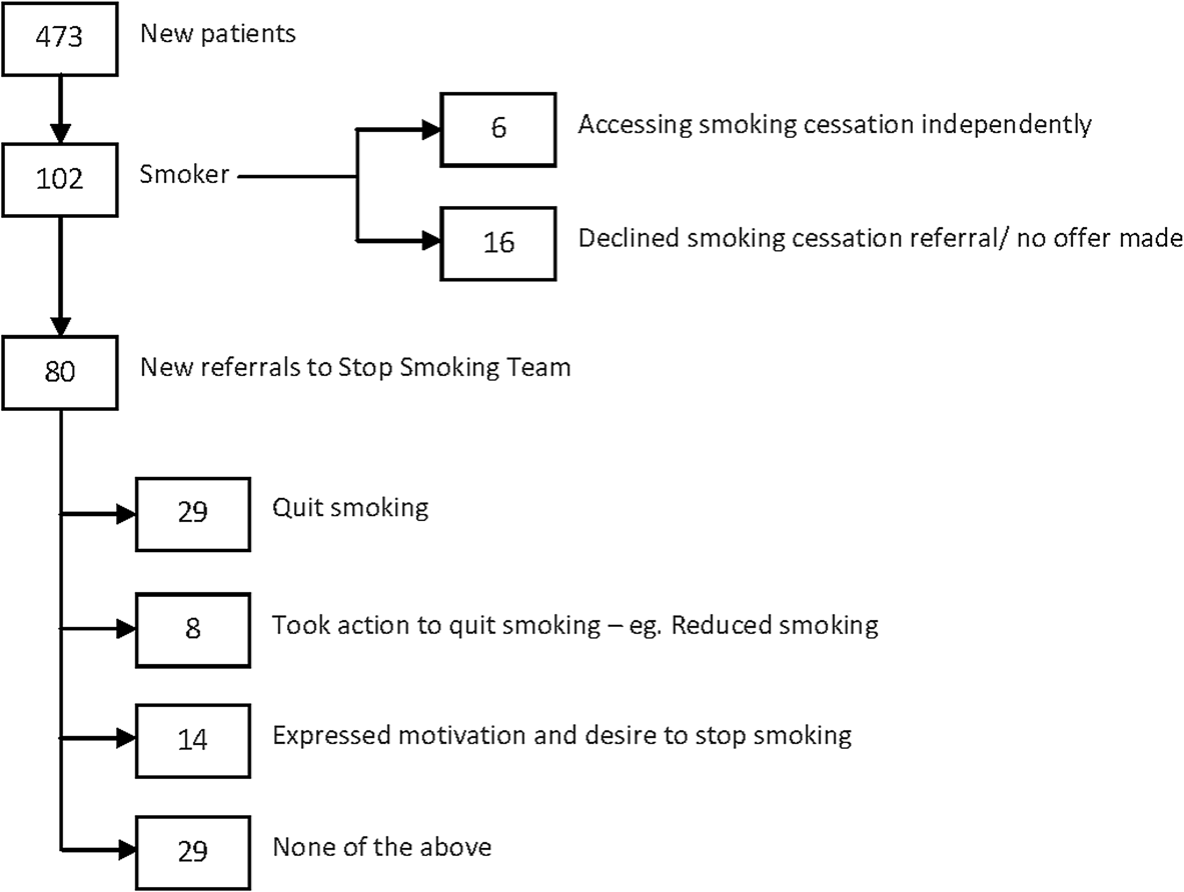
**Flow chart for outcome of smoking cessation intervention.**

A total of 473 patients were seen in the head and neck cancer clinic during the period of study (Figure 1). Of these, 102 (22%) were smokers. Six of the smokers were already attending smoking cessation support and 80 accepted a new referral to the Stop Smoking Service. No offer was made to eight patients as it was deemed inappropriate and eight declined a referral.

**Figure 2:**
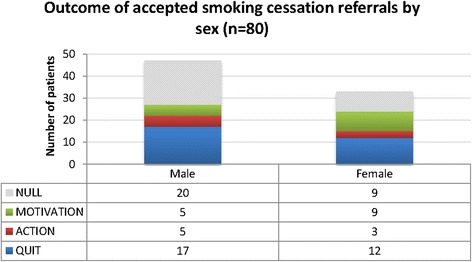
**Outcome of accepted smoking cessation referrals by sex (source: data combined from patient survey and stop smoking team).**

**Figure 3:**
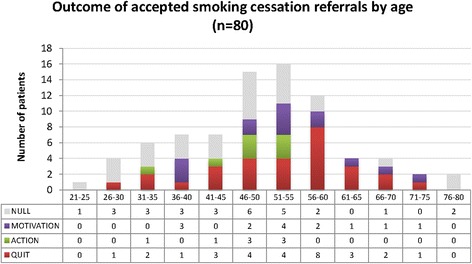
**Outcome of accepted smoking cessation referrals by age (Source: Data combined from patient survey and stop smoking team).**

**Figure 4:**
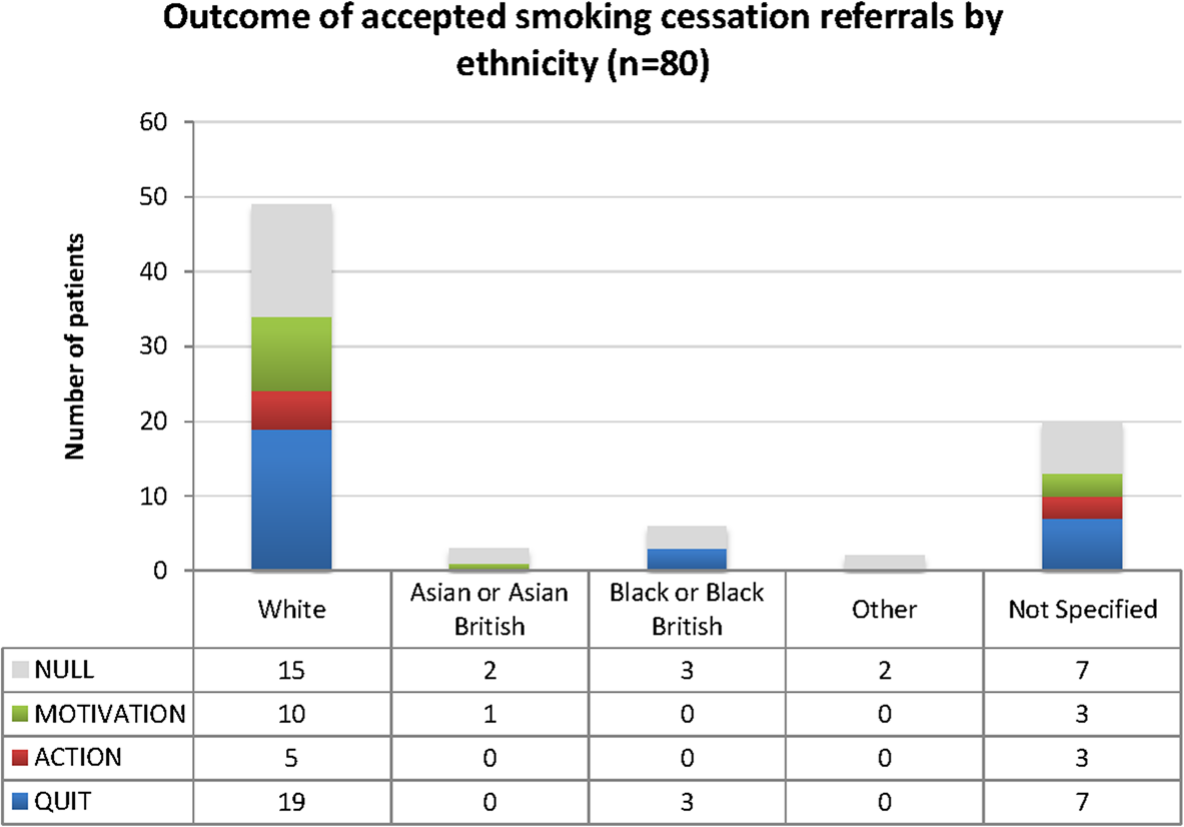
**Outcome of accepted smoking cessation referrals by ethnicity (source: data combined from patient survey and stop smoking team).**

Of the 80 referred patients, 47 (59%) were male and 33 (41%) were female and they covered six decades in age. The 80 new referrals resulted in 29 (36%) patients quitting smoking at least temporarily. Another eight patients reduced their smoking or set a quit date (10%), so the experience of attending the clinic and the intervention impacted favourably on almost half of the patients (46%). Distribution of patient sex, age and ethnicity of patients referred in relation to smoking cessation outcome can be found in Figures 2, 3 and 4. Apart from the extremes of age, there was some evidence of positive change across all groups. Ten out of the 11 patients with a positive diagnosis in this sample provided evidence of quitting.

### Personal interviews

**Figure 5:**
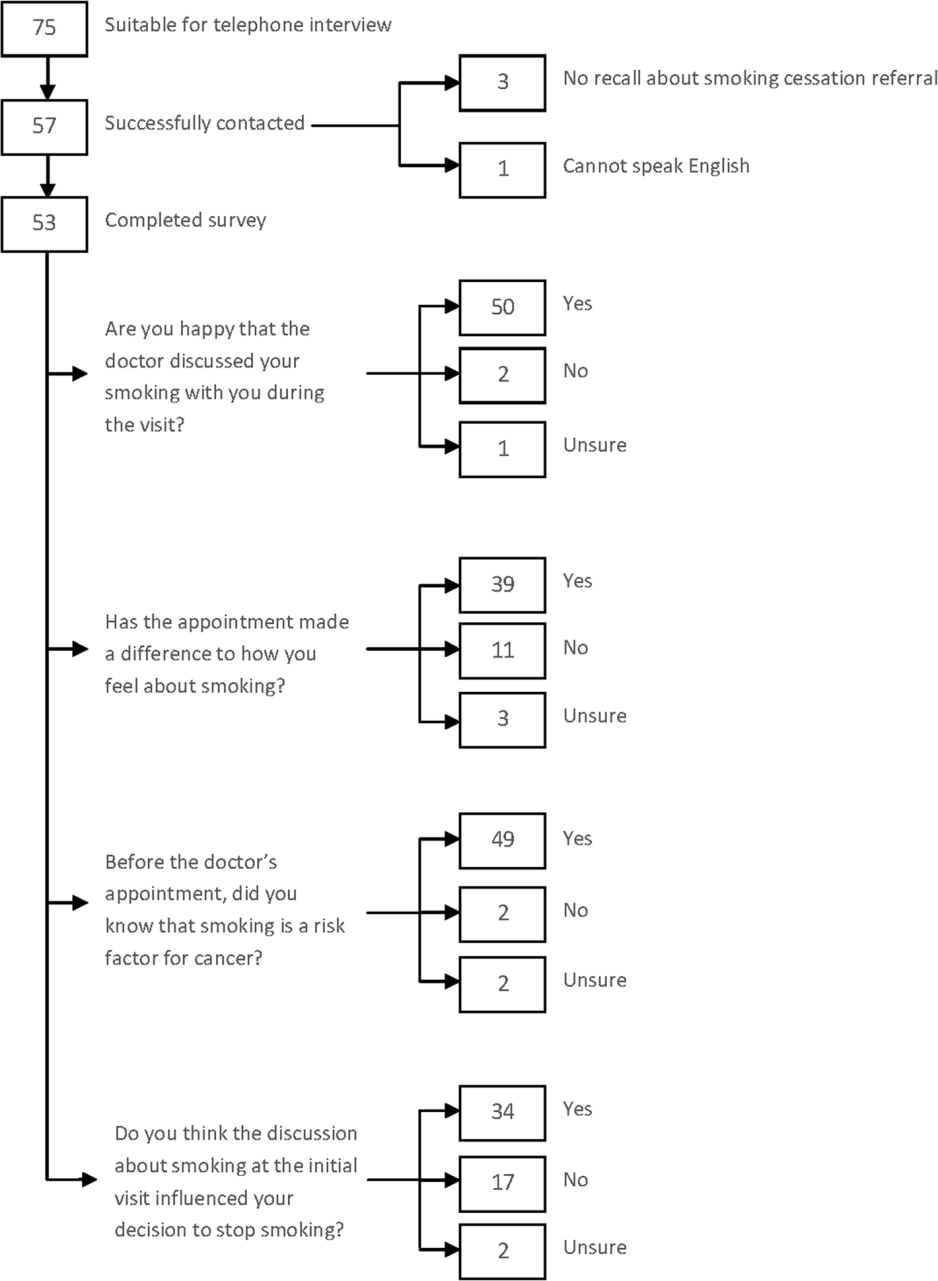
**Flow chart of patient reported outcomes from telephone interview.**

In total, 75 patients were deemed suitable for telephone interview and 57 (76%) were successfully contacted for the telephone survey. Of these 53 (93%) were suitable for completion of the questionnaire. The majority of the respondents (50 out of 53 patients; 94%) were content that the issue of smoking was discussed with the surgeon during their initial consultation visit (Figure 5). Almost three quarters of respondents (n = 39; 74%) reported that the appointment at the head and neck clinic had made a difference as to how they felt about smoking. For some (n = 14; 26%), this was enough to make them quit. When asked whether the discussion about smoking at the initial visit (intervention) influenced their decision to stop smoking, a minority (n = 19; 36%) answered in the negative or were unsure. Four patients (7.5%) further commented that it was the illness itself rather than the intervention that was influential. A small proportion (n = 6; 11%) of patients interviewed indicated that they had no intention to stop smoking.

### Qualitative analysis

There were five elements to the reported satisfaction or dissatisfaction with the intervention:

1. *Nature of the intervention*: Patients felt that the intervention gave them important information and raised awareness about the link between smoking and head and neck cancer. It was also appropriate and relevant to why they were at the appointment. Some however lamented the repetitive nature of intervention as the same message is repeated in various media and settings.
2. *Who delivered the intervention*: Patients highlighted that RO’s position as a consultant surgeon gave him authority as a specialist expert in the field with extensive knowledge and experience with head and neck cancer. Ultimately however, ‘only the smoker can decide’ whether or not to take up the advice.
3. *Timing of the intervention*: Views were divided on whether the timing of the intervention was appropriate. Some patients felt the advice was reassuring and worked as a cue to stop smoking. Other patients felt that it was ‘too much to digest’ in one short appointment, and one patient felt that the consultation ‘was hijacked’ to discuss smoking cessation. Some patients who were diagnosed with head and neck cancer felt that the intervention came too late.
4. *Place of the intervention*: Patients unanimously agreed that the hospital setting is the right place to discuss smoking cessation.
5. *How the intervention was delivered*: Views were divided on whether the method of intervention was appropriate. Patients appreciated the direct and caring nature of RO when delivering the intervention. A minority view, however, was unsure whether the ‘scare tactic’ or ‘shock tactic’ was appropriate. One commented that he felt that the intervention was ‘premeditated and manipulative’ of the patient’s emotions.

## Discussion

The script for intervention in this study was modified from the guidelines on smoking cessation for health professionals [20], published in the British Medical Journal. This brief intervention is divided into four stages: 1. Ask – about smoking status; 2. Advise – all smokers to stop; 3. Assist – the smoker to stop; 4. Arrange – follow up on smoking status or refer for further help. It is not feasible to follow up smokers who reject a smoking cessation referral in a secondary care setting. In this protocol, all patients who declined a smoking cessation referral were given an information leaflet on NHS smoking cessation services so they could access it independently should they change their mind.

Positive behaviour change has been described as a process rather than a distinct event [22]. Various psychological models describe the process as non-linear, as patients move forwards and backwards in the process of change. The stop smoking teams use ‘four week quit’ as a measure of successful smoking cessation. Although this is an arbitrary period, it is useful for measuring outcomes and key performance indicators. In reality, however, most patients quit ‘temporarily’ in the process of change. We therefore made a decision to consider any successful attempts at quitting regardless of time period a positive outcome.

The brief smoking cessation intervention aims to steer patients towards positive behaviour change, which was successful in 46% of patients in this cohort (n = 37). A further 18% (n = 14) of patients were motivated to quit smoking after the appointment. Overall, the findings suggest that the brief intervention made a positive impact on 64% of 80 smokers who accepted a referral to stop smoking team.

Of the 29 patients who quit following the appointment, only 15 (52%) were successfully contacted by the stop smoking team and of these, only seven accepted the service. The brief discussion during the consultation visit was sufficient to support behaviour change for a majority of these patients. The level of cessation related to the head and neck cancer clinic exceeds that for brief simple advice about smoking cessation by medical practitioners [23]. It is likely that the possibility or diagnosis of head and neck cancer had a large influence on patients’ decision to stop smoking. Out of 11 patients who had a positive diagnosis, there was evidence that 10 (91%) quit smoking after the appointment. Although the level of change was higher amongst patients with a positive diagnosis, more of the patients with evidence of positive behaviour change did not have a positive diagnosis at that time.

Despite the overall positive feedback from patients and favourable outcome from the brief intervention, there is still a small group of patients who are resistant to behaviour change. These patients benefit little from smoking cessation advice, advertisements, cigarette packaging regulations, and other tobacco control measures alone. They are aware of the risks of tobacco to health but do not intend to adopt risk reducing behaviours due to other interplaying factors. In this group of patients, combinations of tobacco control measures are needed to encourage behaviour change. Smoking cessation has to be tackled through changes in legislation, economy and culture, and supported by health services. Further research into this group of ‘hard-to-reach’ patients can help develop future plans and guidance for tobacco control.

Limitations of this study include the lack of control group and absence of information about spontaneous smoking cessation and quitting. This was a pilot study conducted within existing resources and further research should include a control group. Another limitation was the variable time interval between the brief intervention and survey interview as patients were not recalled to the clinic and had to be followed up by phone which was subject to patient and researcher availability. This introduces variation in recall and complicates the definition of the outcomes. This was one of the reasons that any attempt at quitting was considered a positive outcome in this analysis, together with the evidence supporting the recognised importance of ‘reduce to quit’ [12,13] in tobacco cessation. Furthermore, patients reported smoking status and quitting could not be validated by urine cotinine levels or carbon monoxide concentrations in expired air [24,25] as further clinical contact at the hospital was not part of their routine care for all patients and this initiative was not resourced for follow up at home. The results from this service evaluation have been presented at King’s Health Partners Cancer Centre biannual research day. Findings of this study are helping to shape policy and inform action on tobacco cessation. This intervention is brief, cheap and evidence-based. Its implementation can be immediate with no need for additional funding. As part of a contemporary health policy, its effects are far reaching beyond the benefits of head and neck cancer prevention in supporting a common risk factor approach.

## Conclusion

The brief smoking cessation intervention during a consultation visit regarding suspected head and neck cancer is an opportunity for health promoting interventions. Within the limitations of this pilot study, it has been found to be effective and acceptable to the majority of the patients surveyed. The prospect or diagnosis of head and neck cancer has a large impact on a patient’s perspective on susceptibility to illness and therefore presents a ‘teachable moment’ where patients are more likely to be motivated to modify behaviour.

### Ethics statement

This study was approved as a service evaluation by the clinical governance team at Guy’s and St Thomas NHS Foundation Trust where this study was carried out. All responses were anonymous and no procedures were performed on humans or animals.

### Availability of supporting data

Please contact the lead author for access to supporting data set.
